# Headache alleviation with nasal irrigation following endoscopic endonasal surgery for pituitary adenomas

**DOI:** 10.1186/s12902-024-01573-w

**Published:** 2024-04-15

**Authors:** Jiayu Gu, Xiaoqun Chen, Xiaoman Cheng, Yunzhi Zou, Zekun Deng, Depei Li, Zhihuan Zhou, Xiaobing Jiang

**Affiliations:** Department of Neurosurgery/Neuro-Oncology, State Key Laboratory of Oncology in South China, Guangdong Provincial Clinical Research Center for Cancer, Sun Yat-sen University Cancer Center, Guangzhou, Guangdong China

**Keywords:** Pituitary adenomas, Endoscopic transsphenoidal surgery, Headache, Sinusitis, Nasal irrigation

## Abstract

**Background:**

Headache is a common occurrence after endoscopic endonasal surgery (EES) for pituitary adenomas and significantly impacts the quality of life of patients. This study aims to investigate the effectiveness of nasal irrigation in relieving postoperative headache after EES.

**Methods:**

A retrospective analysis was conducted on a cohort of 101 patients (Cohort I) who underwent EES for pituitary adenomas to explore the risk factors associated with postoperative headache. Another cohort of 72 patients (Cohort II) who received adjuvant nasal irrigation following surgery was enrolled for further analysis. The Headache Impact Test (HIT-6) was used to score the severity of headache, and patients with a HIT score > 55 were classified as having headache.

**Results:**

In Cohort I, 21.78% of patients experienced headache one month after EES, which decreased to 5.94% at the three-month follow-up. Multivariate analysis revealed that postoperative nasal sinusitis (OR = 3.88, 95%CI 1.16–13.03, *p* = 0.028) and Hardy’s grade C-D (OR = 10.53, 95%CI 1.02-109.19, *p* = 0.049) independently predicted the presence of postoperative headache at one month. At the three-month follow-up, patients with sinusitis had higher HIT-6 scores compared to those without sinusitis (44.43 ± 9.78 vs. 39.72 ± 5.25, *p* = 0.017). In Cohort II, the incidence of sinusitis at three months was significantly lower than that in Cohort I (*p* = 0.028). Importantly, both the incidence of headache and HIT-6 scores in Cohort II were significantly lower than those in Cohort I at the one- and three-month follow-ups.

**Conclusions:**

Postoperative sinusitis is an independent risk factor for the development of headache following EES for pituitary adenomas. Prophylactic nasal irrigation helps relieve postoperative headache, possibly by preventing the occurrence of sinusitis.

## Introduction

Pituitary adenomas (PAs) are among the most common primary tumors of the central nervous system, accounting for 10–15% of all intracranial tumors [1–3]. At present, transsphenoidal surgery is the primary treatment for most PAs [4]. Endoscopic endonasal surgery (EES) has shown advantages in removing pituitary adenomas, and become the mainstream choice [5–7]. In the long time, complications with endoscopic transsphenoidal surgery include epistaxis, sinusitis, hyposmia and hypopituitarism [8, 9]. Chronic headache is also very frequent postoperatively, and affects the quality of life of patients. The incidence of headaches in patients with pituitary adenomas has been reported to range between 33% and 72% [10–12], and only half of them were relieved postoperatively, although the tumor has been removed [13]. The reasons underlining the pre- and postoperative headache should be different, and the treatment strategy is also different.

Previous studies tried to investigate the potential risk factors associated with chronic headache post transsphenoidal surgery. Headache associated with pituitary adenomas may be induce through displacement of intracranial pain-sensitive structures located in the blood vessels, cranial nerves and dura mate, inflammation involving pain-sensitive structures or meningeal irritation, or hormonal dysregulation [12, 14]. Additionally, hypothalamic regulation has been identified to play a crucial role in regulating headache. Therefore, larger tumor volume, cavernous sinus invasion, optic chiasm compression and functioning PAs were shown to be independent risk factors [12, 15]. At the same time, F Sireci et al. reported that low-dose clarithromycin may help to improve patient complaints after EES [16]. However, some studies reported the negative results, where cavernous sinus invasion, suprasellar extension, and optic chiasm compression were not associated with the incidence of postoperative headache [14, 17]. Several factors may account for the inconsistence of previous studies. Firstly, both groups of patients treated by transsphenoidally microsurgical approach and EES were enrolled for analysis, which might induce the variation of conclusions from different studies [18, 19]. Secondly, the evaluation scale for headache varied among the studies [12, 14, 17–19]. Moreover, the way of seller re-construction during ESS will also induce various extent of pain [20]. Last and most importantly, the evaluation time was also different among the studies, and few studies have evaluated the headache dynamically.

To address these limitations, we enrolled a cohort of 101 patients with PAs who underwent EES exclusively to investigate the risk factors associated with chronic headache. In order to comprehensively assess the profile of headache risk factors, we conducted a dynamic evaluation of headache at the preoperative stage, as well as at 1 month and 3 months postoperatively. Our findings demonstrated that prophylactic nasal irrigation following EES could effectively reduce the incidence of sinusitis and postoperative headache.

## Patients and methods

### Population and data collection

For this study, we conducted a retrospective review of two consecutive cohorts of patients. The first cohort, Cohort I, consisted of 101 patients who underwent EES at Sun Yat-sen University Cancer Center (SYSUCC) between July 2018 and December 2020. This cohort was enrolled to investigate the risk factors associated with postoperative headache. The second cohort, Cohort II, included 72 patients who received care with nasal irrigation following EES between January 2021 and November 2021. This cohort was specifically enrolled to study the effect of nasal irrigation on postoperative headache. The study received ethical approval from the Research Ethics Committee of SYSUCC (2021-FXY-235), and all patients were informed about the study and provided their consent to participate.

Inclusion criteria for the study involved patients who underwent EES for pituitary adenoma removal, had confirmed pathology, and complete follow-up data. Patients who underwent craniotomy resection for pituitary adenomas or those with incomplete follow-up data were excluded from the study.

The EES resection of PAs was carried out by an experienced neurosurgeon. Typically, the procedure involved performing a posterior nasal septectomy to allow binasal access. The face of the sphenoid and intersphenoidal septations were removed. After incising the dura, the tumor was resected. In cases of complex intraoperative cerebrospinal fluid leaks, a layered repair approach was employed. This involved using a combination of materials such as Gelfoam, fat, fascia, bone, and a vascularized nasoseptal flap. Additionally, a catheter balloon was placed in the posterior nostril for reconstruction purposes.

All patients underwent preoperative CT plain scan and contrast-enhanced MRI of the head. Clinical data, including age, gender, pituitary apoplexy, diameter of tumor, clival osseous destruction, sinusitis, extent of tumor resection and postoperative intracranial hemorrhage, were collected. Nasal sinusitis was defined by an experienced radiologist according to the enhanced MRI, where marked hyperintensity on T2-weighted (T2W) MRI images and enhancement of mucosal thickening on enhanced T1-weighted (T1W) MRI images [21, 22]. The invasiveness of PAs was graded with Knosp’s [23] and Hardy’s [24] grade system. The Headache Impact Test (HIT-6) was used to evaluate the extent of headache preoperatively, 1 month and 3 months postoperatively [25, 26]. Patients were with a HIT-6 scores of > 55 was considered to have headache [27, 28].

### Nose care during the follow-up of patients post-EES for PAs

A follow-up MRI will be performed 1 month, and 3 months postoperatively to assess residual tumor and sinusitis. Patients in cohort II will be suggested to start nose rinsing every two days for 2 months, by using a nasal irrigator filled with saline. Before discharging, those patients will be taught how to use the irrigator. In addition, they will also get an instruction detailed the rising techniques. After discharging, all the patients will be tightly followed up in a Wechat group.

### Statistical analysis

Data analysis was performed using SPSS version 20.0 (IBM Corp.). Quantitative data were presented as mean ± standard deviation (SD). To determine statistically significant differences between the two groups, independent sample t-tests and Pearson chi-square tests or Fisher’s exact tests were employed. Univariate and multivariate analyses were conducted to analyze the risk factors associated with headache. A p-value of less than 0.05 was considered statistically significant.

## Results

### Basic clinical and pathological characteristics of the two cohorts of patients

In this study, we initially enrolled a cohort of 101 patients (Cohort I) to investigate the risk factors associated with postoperative headache following EES for pituitary adenomas. Subsequently, another cohort of pituitary adenoma patients (Cohort II) who received nasal irrigation after EES was enrolled to validate the effectiveness of nasal irrigation in relieving postoperative headache. Table 1 presents the comparable basic clinical and pathological characteristics between the two cohorts. Overall, the mean maximum diameter of the tumors was 28.24 ± 10.61 mm. Among the patients, 77 (44.5%) had tumors classified as Knosp’s grade III-IV, and 16 patients had tumors classified as Hardy’s Grade C-D. Gross total resection was achieved in 152 patients (87.9%). Among the cases, 50 (28.9%) were functioning pituitary adenomas, with 34 of them being growth hormone-secreting adenomas. Additionally, 47 samples (27.2%) exhibited positive staining for the transcription factor TBX19. The average Ki-67 index value was 2.38 ± 1.56. Preoperatively, 56 patients (32.4%) were diagnosed with mild sinusitis based on head MRI findings.

### Preoperative headache was significantly relived 3-month after operation

**Fig. 1:**
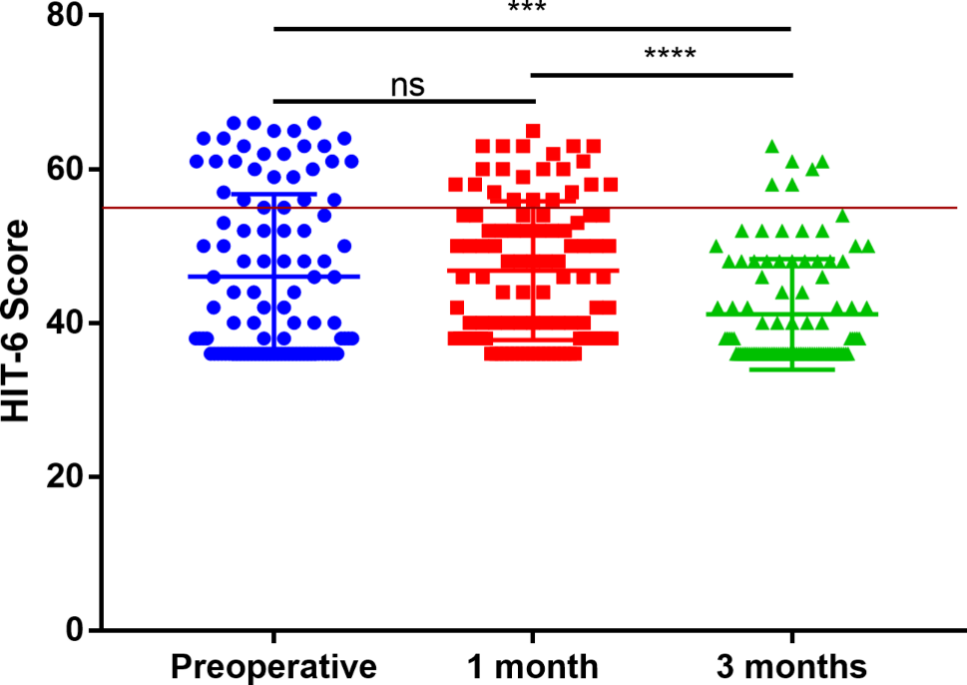
Scatter plot of HIT-6 scores preoperatively, 1 or 3 months postoperatively in Cohort I (Horizontal red line represents HIT-6 scores = 55, similarly hereinafter in Figs. 2 and 4)

In Cohort I, a total of 26 patients (25.7%) experienced headaches preoperatively, and this number decreased to 22 patients (21.8%) at 1 month postoperatively. Furthermore, only 6 patients still reported headaches at the 3-month postoperatively. Overall, the HIT-6 scores at 3 months postoperatively were significantly lower compared to the preoperative scores (41.12 ± 0.72 vs. 46.06 ± 1.06, *p* = 0.0002) and the 1-month postoperative scores (41.12 ± 0.72 vs. 46.82 ± 0.90, *p* < 0.0001) (Fig. 1). However, there was no statistically significant difference between the preoperative HIT-6 scores and the scores at 1 month postoperatively. These findings suggest that patients who experienced headaches preoperatively experienced significant relief three months after the operation.

**Table 1:** Clinicopathological features of patients underwent EES for pituitary adenoma between with and without nasal irrigation groups (*N*=173)

	N (%)	Without nasal irrigation (n=101)	With nasal irrigation (n=72)	*P value*
Gender				0.464
Male	88(50.9%)	49	39	
Female	85(49.1%)	52	33	
Tumor apoplexy				0.126
Yes	25(14.5%)	20	5	
No	148(85.5%)	81	67	
Preoperative sinusitis				0.819
Yes	56(32.4%)	32	24	
No	117(67.6%)	69	48	
Clival osseous destruction				0.130
Yes	17(9.8%)	7	10	
No	156(90.2%)	94	62	
Extent of tumor resection				0.077
Total resection	152(87.9%)	85	67	
Subtotal resection	21(12.1%)	16	5	
Intraoperative cerebrospinal fluid leak				0.912
Yes	44(25.4%)	26	18	
No	129(74.6%)	75	54	
Postoperative sinusitis				0.418
Yes	88(50.9%)	54	34	
No	85(49.1%)	47	38	
Knosp’s grade				0.065
Grade 0-2	96(55.5%)	62	34	
Grade 3-4	77(44.5%)	39	38	
Hardy’s grade				0.075
Grade A, B&E	157(90.8%)	95	62	
Grade C-D	16(9.2%)	6	10	
Functioning pituitary adenoma				0.077
Yes	50(28.9%)	24	26	
No	123(71.1%)	77	46	
GH secreting adenoma				0.060
Yes	34(19.7%)	15	19	
No	139(80.3%)	86	53	
TBX19				0.233
positive	47(27.2%)	24	23	
negative	126(72.8%)	77	49	
Age (years; mean±SD)	46.78±12.42	45.71±11.90	48.28±13.04	0.181
Maximum diameter (mm; mean±SD)	28.24±10.61	28.35±11.04	28.10±10.06	0.879
Ki-67 index (%;mean±SD)	2.38±1.56	2.56±1.64	2.11±1.40	0.059

### Postoperative sinusitis independently predicts the presence of postoperative headache

To compare the risk factor patterns of pre- and post-operative headaches, we first analyzed the preoperative HIT-6 scores. Univariate and multivariate analyses revealed that both pituitary apoplexy (odds ratio [OR] = 3.59, 95% confidence interval [CI]: 1.22–10.58, *p* = 0.020) and Hardy’s grade C-D (OR = 21.06, 95% CI: 2.25-197.02, *p* = 0.008) were independent risk factors for preoperative headaches (Table 2).

**Table 2:** Multivariate analysis of risk factors of preoperative headache in pituitary adenoma patients (*N*=101)

	Univariate logistic regression analysis					Multivariate logistic regression analysis				
	OR	95% CI	*P value*	B value			OR	95% CI	*P value*	
Preoperative sinusitis	0.737	(0.274, 1.984)	0.546	-			-	-	-	
Clival osseous destruction	4.364	(0.907, 21.001)	0.066	-			-	-	-	
Knosp’s grade	0.991	(0.396, 2.479)	0.985	-			-	-	-	
GH secreting adenoma	0.397	(0.083, 1.894)	0.247	-			-	-	-	
TBX19	1.257	(0.453, 3.491)	0.661	-			-	-	-	
Tumor apoplexy	3.080	(1.099, 8.633)	0.032	1.278			3.591	(1.219, 10.575)	0.020	
Hardy’s grade	17.619	(1.950, 159.178)	0.011	3.048			21.064	(2.252, 197.023)	0.008	

Next, we examined the risk factors associated with headaches at 1 month postoperatively. As shown in Table 3, the risk factor pattern differed from the preoperative analysis. Hardy’s grade C-D (OR = 10.53, 95% CI: 1.02-109.19, *p* = 0.049) remained an independent risk factor for postoperative headaches. Additionally, postoperative sinusitis (OR = 3.88, 95% CI: 1.16–13.03, *p* = 0.028) was found to independently predict the presence of headaches at 1 month postoperatively. Moreover, the proportion of sinusitis among patients with headaches was higher than that among patients without headaches at 3 months postoperatively (100.0% vs. 25.3%, *p* < 0.001).

**Table 3:** Risk factor analysis of postoperative headache in pituitary adenoma patients 1 month after EEA (*N*=101)

	Univariate logistic regression analysis					Multivariate logistic regression analysis			
	OR	95% CI		*P value*	B value		OR	95% CI	*P value*
Tumor apoplexy	0.875	(0.260, 2.947)		0.829				-	-
Preoperative sinusitis	1.008	(0.365, 2.781)		0.988	-		-	-	-
Knosp’s grade	0.885	(0.332, 2.355)		0.806	-		-	-	-
GH secreting adenoma	1.374	(0.391, 4.827		0.620	-		-	-	-
TBX19 positive	1.271	(0.434, 3.725)		0.662	-		-	-	-
Clival osseous destruction	5.63	(1.156, 27.404)		0.032					
Postoperative sinusitis	5.375	(1.668, 17.325)		0.005	1.357		3.883	(1.157, 13.029)	0.028
Hardy’s grade	22.941	(2.516, 209.166)		0.005	2.354		10.53	(1.015, 109.189)	0.049

**Fig. 2:**
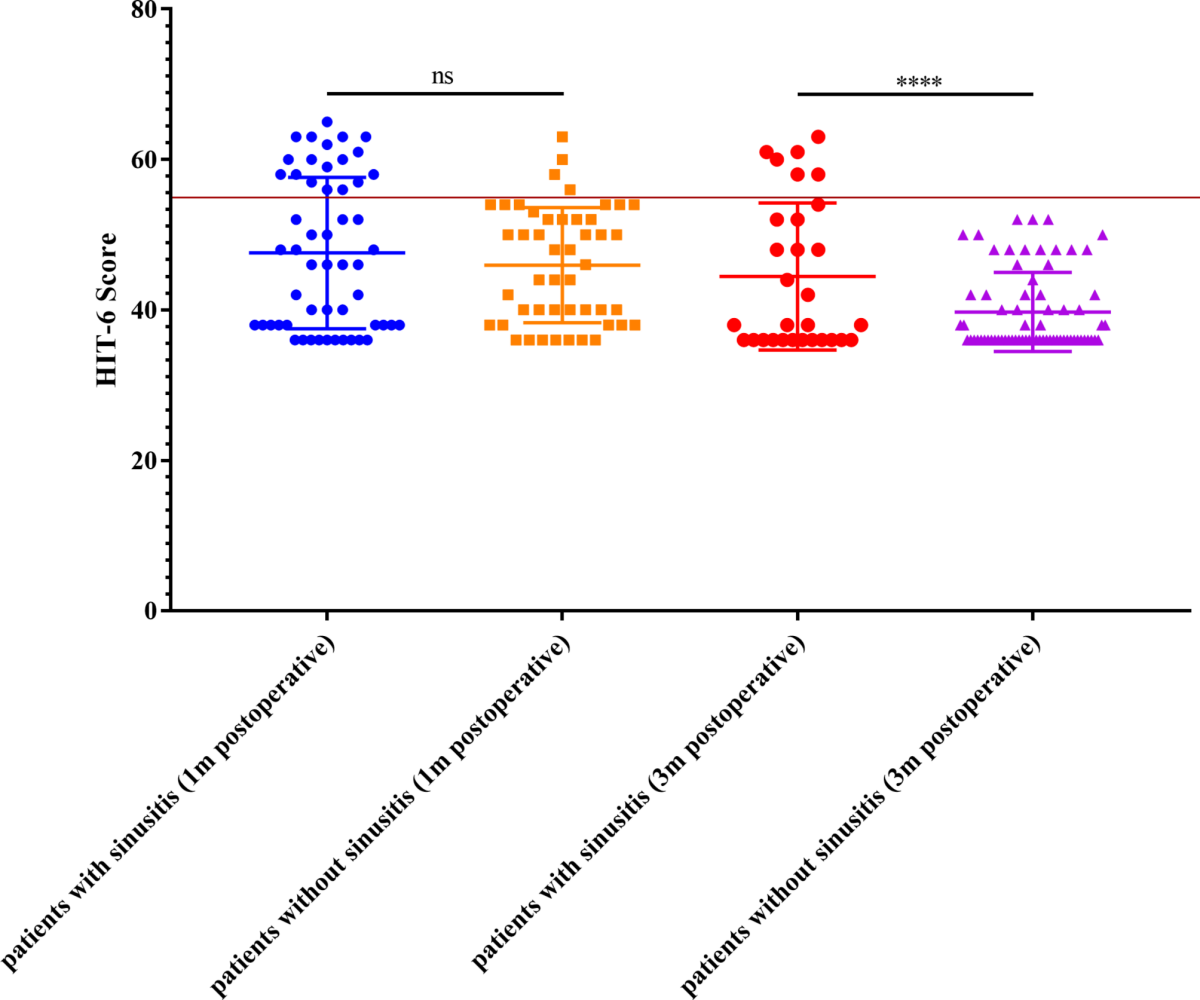
Scatter plot of HIT-6 scores of patients with/without sinusitis 1 or 3 months postoperatively

Lastly, we investigated the relationship between postoperative sinusitis and headaches in the subgroup of patients with postoperative headaches. Out of the 22 patients who experienced headaches at 1 month postoperatively, the proportion of sinusitis was significantly higher in the headache group compared to the non-headache group (81.8% vs. 45.6%, *p* = 0.006). At 3 months, 6 patients still had headaches, and all of them were confirmed to have postoperative sinusitis. Furthermore, the 3-month HIT-6 scores of patients with sinusitis were significantly higher than those of patients without sinusitis (44.43 ± 9.78 vs. 39.72 ± 5.25, *p* = 0.017) (Fig. 2). Overall, these data indicate a strong association between postoperative sinusitis and headaches.

### Adjuvant nasal irrigation helps to decrease the presence of postoperative sinusitis and headache

We conducted an analysis to investigate the effect of nasal irrigation on postoperative headache in Cohort II. Nasal saline irrigation has previously been shown to alleviate nasal sinusitis [29–30]. We hypothesized that preventing sinusitis through nasal irrigation could help reduce the presence of postoperative headaches following EES for pituitary adenomas.

**Fig. 3:**
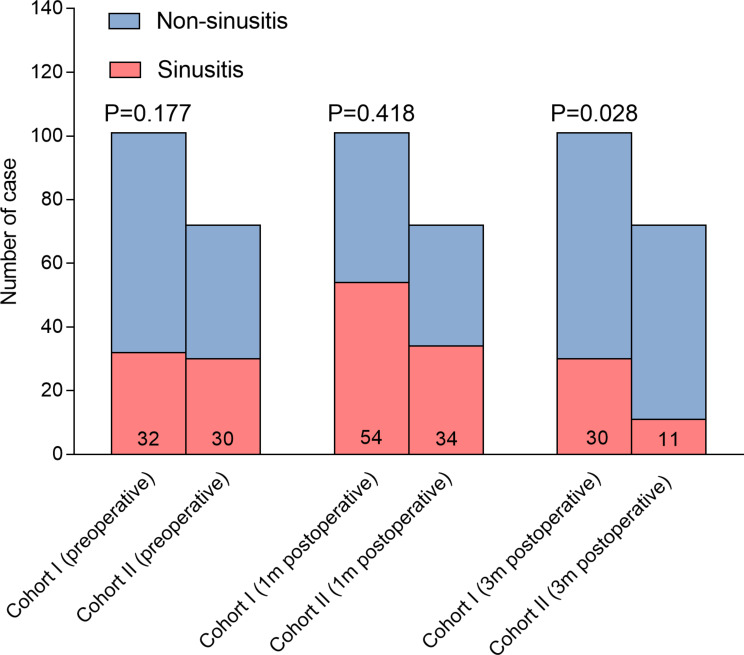
Bar-Stacked segmentation depicting the composition of sinusitis and p value in Pearson chi-square test in non-nasal irrigation group and nasal saline irrigation group

**Fig. 4:**
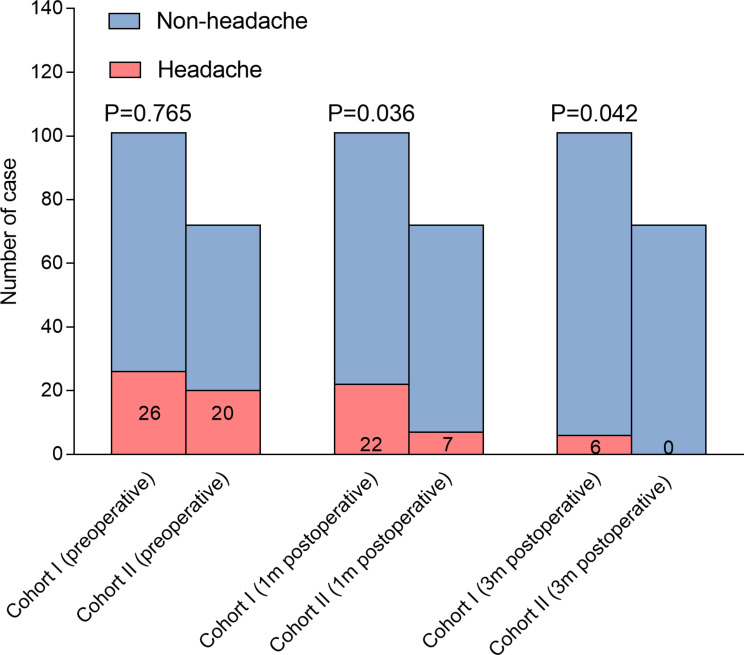
Bar-Stacked segmentation depicting the composition of headaches measured by the HIT-6 questionnaire and p value in Pearson chi-square test in non-nasal irrigation group and nasal saline irrigation group

**Fig. 5:**
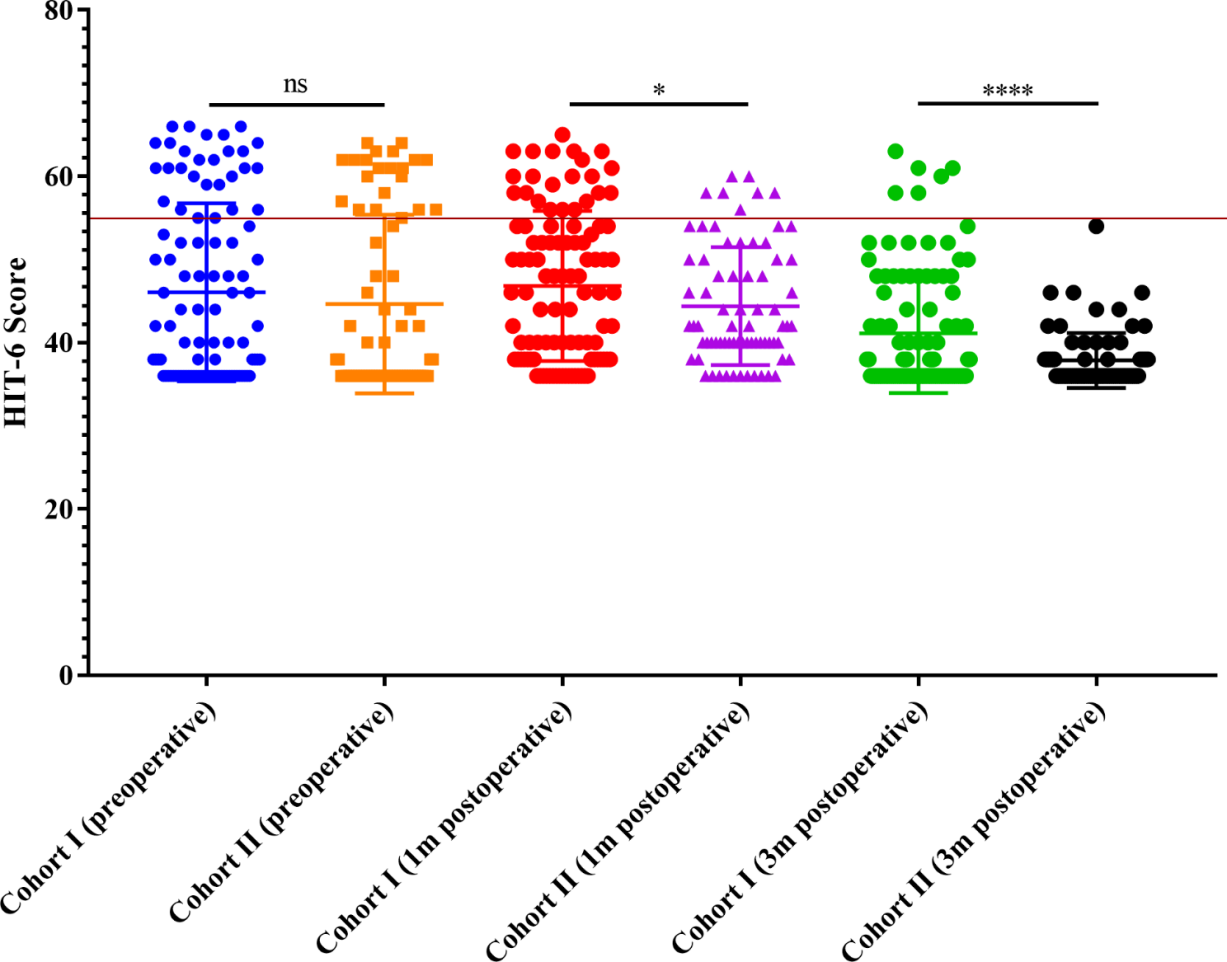
Scatter plot of HIT-6 scores in non-nasal irrigation group and nasal saline irrigation group

In Cohort II, the incidence of sinusitis at 3 months was significantly lower compared to Cohort I (Fig. 3). Importantly, the proportion of headaches was significantly lower in Cohort II compared to Cohort I at both 1- and 3-month follow-ups (Fig. 4). Consistently, the HIT-6 scores at 1 month (44.39 ± 7.09 vs. 46.82 ± 9.02, *p* = 0.049) and 3 months (37.86 ± 3.31 vs. 41.12 ± 7.19, *p* < 0.001) in Cohort II were significantly lower compared to those in Cohort I (Fig. 5). These findings suggest that adjuvant nasal irrigation may help alleviate headaches following EES for pituitary adenomas. The lower incidence of sinusitis and improved headache outcomes in Cohort II support the potential benefit of nasal irrigation in preventing postoperative headaches.

## Discussion

Chronic headache is a common complication following transsphenoidal resection of pituitary adenomas (PAs). However, the incidence and risk factors associated with this complication have shown inconsistency in previous studies. By evaluating headaches at multiple time points, we identified a distinct pattern of risk factors for headaches before and after the operation. This finding may help explain the inconsistent conclusions reported in prior research. Notably, our study revealed that postoperative sinusitis, rather than preoperative sinusitis, was an independent risk factor for the occurrence of headaches. This finding is significant as it highlights the importance of implementing measures to prevent postoperative sinusitis, which may subsequently reduce the occurrence of postoperative headaches.

To assess whether hypothalamus repression could lead to headaches, we categorized patients into two groups based on Hardy’s grade: Hardy’s grade C-D and Hardy’s grade A, B, and E. As indicated in Table 1, the proportion of patients with headaches was significantly higher in the Hardy’s grade C-D group compared to the other group (22.7% vs. 1.3%, *p* = 0.002). Furthermore, in multivariate analysis, Hardy’s grade C-D independently predicted the presence of headaches in both the preoperative and postoperative analyses. Previous studies have demonstrated the involvement of hypothalamic regulation in migraine and cluster headaches. Additionally, a structure in close proximity to the hypothalamus has been identified as playing a crucial role in the generation of these headache attacks [31].

The cavernous sinus contains structures that can generate pain, such as the internal carotid artery and the trigeminal nerve and ganglion. The invasion of these structures might be expected to cause pain. However, different studies have presented varying opinions on whether cavernous sinus invasion leads to pain [12, 14, 17]. The inconsistency in findings could be attributed to differences in the timing of headache evaluation and the scales used to assess pain. In our study, we evaluate the risk factors at multiple points, including preoperative, 1 month and 3 months postoperatively. Knosp’s grade was not shown to be significantly associated with headache at all points. Much more deliberated studies are warranted to declare their correlations.

Postoperative sinusitis is more common due to the opening of the sinuses in the nasal cavity and the need to open the sphenoid sinus for endoscopic resection of pituitary adenomas [32]. Sinusitis itself can cause headaches [33]. Given the high sensitivity of the nasal mucosa, the sensation of pain is more noticeable after surgery via endoscopic transsphenoidal surgery. In our study, preoperative sinusitis was determined by craniocerebral MRI in 31.7% of patients, and the proportion of postoperative sinusitis increased to 53.5%. The proportion of postoperative sinusitis was higher in the headache group compared to the non-headache group. Multivariate logistic regression analysis showed that the odds ratio (OR) of postoperative headache in patients with postoperative sinusitis was 3.883. When analyzing data of headache and sinusitis, we found that the proportion of sinusitis in patients with postoperative headache at the same period is always higher than that without headache. These findings indicate a strong association between postoperative sinusitis and headaches, suggesting that treating sinusitis may help prevent the occurrence of postoperative headaches.

In some studies, patients with functioning pituitary adenomas are more likely to develop postoperative headaches [14], especially nociceptive tumors like growth hormone secreting tumors and prolactinomas. These headaches may potentially respond to endocrinological treatments [34, 35]. However, in our study involving patients with pituitary adenomas, we did not find an association between functioning pituitary adenomas and postoperative headaches. We suspect that some patients with functioning pituitary adenomas have achieved biochemical remission after surgery, leading to a decrease in hormone levels to normal or near-normal levels, which may explain the absence of headaches. It is necessary to further verify the relationship between postoperative hormone levels and headache. In addition, nasal packing and dissection of a naso-septal flap may also cause postoperative headache. However, multilayer repair was not a risk factor for both short-term and long-term postoperative headache.

Our study also has some limitations. Firstly, the sample size of this study is still insufficient and the follow-up time is limited. It is necessary to further increase the sample size and extend the follow-up time to investigate the risk factors of long-term headache. Secondly, we failed to evaluate the status of pituitary function comprehensively, which is an important aspect of patients’ quality of life. In addition, rhinosinusitis was merely defined by enhanced MRI, not including symptoms and nasal endoscopy. A more comprehensive evaluation of rhinosinusitis, including clinical symptoms and nasal endoscopy, would provide a more accurate assessment of its association with postoperative headaches. Finally, the causation relationship between sinusitis and postoperative headache remains unknown. Prospective, randomized studies comparing treatment and no treatment of sinusitis are needed to establish a clearer understanding of this relationship.

In conclusion, headache is a very common complication following EES for pituitary adenomas. Postoperative sinusitis and Hardy’s grade are independent risk factors for postoperative headache. Prophylactic treatments may help to prevent postoperative sinusitis, and thus reduce the incidence of headache. More prospective clinical studies including more patients are suggested to confirm our findings.

## Data Availability

Related data and materials could be retrieved by requiring corresponding authors.
